# The Uniform System of Accounts

**Published:** 1904-02-27

**Authors:** 


### THE UNIFORM SYSTEM OF ACCOUNTS.

B. R. I.—Account keeping is notoriously better in the North than in the South. The nearest hospitals for you are the Royal Devon and Exeter Hospital, Exeter, and the Swansea General Hospital, both of which publish their accounts on this system.

